# *miR-200a-3p* Regulates PRKACB and Participates in Aluminium-Induced Tau Phosphorylation in PC12 Cells

**DOI:** 10.1007/s12640-022-00609-0

**Published:** 2022-12-02

**Authors:** Huan Li, Qun Liu, Qinli Zhang, Xingli Xue, Jingsi Zhang, Jing Zhang, Li Lin, Qiao Niu

**Affiliations:** 1 Department of Occupational Health, School of Public Health, Jining Medical University, Jining, 272067 Shandong China; 2 Department of Occupational Health, School of Public Health, Shanxi Medical University, Taiyuan, 030001 Shanxi China; 3 Department of Occupational Health, School of Public Health, Xuzhou Medical University, Xuzhou, 221000 Jiangsu China; 4 Key Lab of Environmental Hazard and Health of Shanxi Province, Shanxi Medical University, Taiyuan, 030001 Shanxi China; 5 Key Lab of Cellular Physiology of Education Ministry, Shanxi Medical University, Taiyuan, 030001 Shanxi China; 6 Department of Occupational Health, School of Public Health, Binzhou Medical University, Binzhou, 256600 Shandong China

**Keywords:** Aluminium, miR-200a-3p, PRKACB, p-tau, CREB

## Abstract

Aluminium (Al) is an environmental neurotoxin that humans are widely exposed to, but the molecular mechanism of its toxic effects is not fully understood. Many studies have shown that exposure to Al can cause abnormal phosphorylation of the tau protein that is believed as one of pathological features of Alzheimer’s disease. Increasing evidence indicates that microRNAs (miRNAs) may be involved in the pathological processes of neurodegenerative diseases and are potential regulatory factors for related target genes. Phosphorylation at Ser-133 of cAMP response element-binding protein (CREB) is one of the major pathways of CREB activation, and phosphorylation at this site is controlled by protein kinase A (PKA). The catalytic subunit of PKA, cAMP-dependent protein kinase catalytic subunit beta (PRKACB), phosphorylates CREB. The target gene prediction software TargetScan showed that PRKACB was one of the target mRNAs of miR-200a-3p. The purpose of this study was to investigate whether miR-200a-3p regulates the PKA/CREB pathway by targeting PRKACB and leads to abnormal phosphorylation of the tau protein in nerve cells. The results showed that Al exposure increased the expression level of miR-200a-3p, and miR-200a-3p increased the expression of targeted downregulated PRKACB, and then decreased the PKA/CREB signalling pathway activity, leading to abnormal hyperphosphorylation of tau.

**Graphical Abstract:**

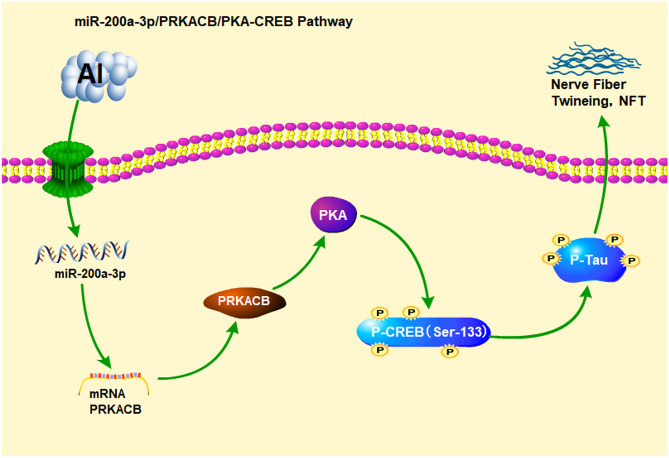

## Introduction

Aluminium (Al), the most abundant metal element in the Earth crust (Bondy 2010), is a widely occurring environmental neurotoxin, and a large amount of Al exists in the living environment of humans. Al was previously thought to be a non-toxic metal until the 1970s, when it was recognised to cause a variety of diseases (Klein 2005). Owing to the superior performance of Al, it is widely used in production and life; thus, humans come into contact with Al through a variety of ways, such as food additives, household appliances, and biomedical fields (Krewski et al. 2007), and it can enter the human body through drinking water, food, and medicine (Stahl et al. 2017). Several studies have shown that it is closely related to a variety of neurodegenerative diseases, such as Alzheimer’s disease (AD) and Parkinson’s disease (PD). However, the molecular mechanism of its toxicity is not completely understood and its neurotoxic mechanism requires further study (Exley et al. 2012; Yasui et al. 1992; Alfrey et al. 1976). There are two signature pathological features of AD: senile plaques and neurofibrillary tangles (NFT). Some studies suggest that NFT is a sign of early brain ageing and the main cause of neuronal degeneration (Peethumnongsin et al. 2010). There is a highly significant correlation between the number of NFT and the severity of AD (Wilcock and Esiri 1982). NFT is mainly caused by hyperphosphorylation of the tau protein (Torreilles and Touchon 2002). Both neurotoxic Aβ and hyperphosphorylated tau protein increase the level of pro-inflammatory factors in the brain, resulting in neuronal damage or death, and further activation of glial cells, leading to harmful cycles of neuroinflammation and neurodegeneration (Leyns and Holtzman 2017).

In normal brain tissues, the cellular function of tau protein is to bind to tubulin and maintain microtubule stability (Mandelkow and Mandelkow 2012; Drechsel et al. 1992; Weingarten et al. 1975). However, tau protein in the brains of AD patients is abnormally phosphorylated, separated from microtubules, and aggregated into pairs of spiral filaments, which exist in nerve fibres and malnourished neurites. Finally, it engages with straight filaments, forms NFT, and loses its normal function, resulting in decreased neuronal activity and affecting cognitive ability (Drummond et al. 2020). Accumulation of phosphorylated tau protein (p-tau) is closely related to synaptic damage, neurodegeneration, and the development of dementia. Studies have shown that Al is closely related to abnormal phosphorylation of the tau protein, and can cause pathological reactions such as abnormal phosphorylation of the tau protein (Walton 2007, 2006; Wang et al. 2021).

Non-coding RNAs (ncRNAs) are particularly abundant in the central nervous system. Recent studies suggest that there may be a close relationship between ncRNAs and AD, and changes in their expression may be related to brain ageing and neurodegenerative diseases (Idda et al. 2018). Studies have shown that ncRNA and their target messenger RNA are abnormally expressed in the central nervous system, cerebrospinal fluid, and serum of patients with AD, suggesting that they are involved in the pathogenesis of AD (Takousis et al. 2019). MicroRNA (miRNAs) are single-stranded ncRNA molecules with lengths of approximately 18–22nt. miRNAs inhibit gene expression at the post-transcriptional level by pairing with the 3′ untranslated region (3′ UTR) of target mRNA and affect the stability and translation of mRNA (Gao et al. 2010; Hou et al. 2011; Banks et al. 2020). miRNAs have been shown to regulate a variety of biological and cellular processes related to various human diseases (Izaurralde 2015), and play an important role in the occurrence and development of various diseases (Kang 2017; Li et al. 2020), with specificity for tissues, cells, and diseases (Ratnadiwakara et al. 2018).

miRNAs play a broad regulatory role in many aspects of the nervous system by participating in the post-transcriptional regulation of gene expression in multicellular organisms. Studies have shown that abnormal expression of miRNAs is associated with a variety of neurological diseases, such as neurodegenerative diseases such as AD (Basak et al. 2016). With the deepening of relevant studies, more and more evidences indicate that miRNA may be involved in the pathological process of AD and is a potential regulatory factor of related target genes in AD (Liu et al. 2014; Li et al. 2021a). miR-200a-3p belongs to the miR-200 family, which plays an important role in many human diseases (such as cancer and AD) and regulates cell differentiation, apoptosis, and proliferation (Feng et al. 2014). There is an obvious debate regarding the levels of miR-200a-3p detected in various AD models, and studies have shown that miR-200a-3p expression is downregulated during AD progression (Liu et al. 2014). However, some experiments have shown that the expression of miR-200a-3p is increased in the hippocampus of patients with AD and APP/PS1 mice (Zhang et al. 2017; Lau et al. 2013). It was found that miR-200a-3p has multiple downstream targets using the target gene prediction software TargetScan, which shows the complexity of the specific role and potential molecular mechanism of miR-200a-3p in AD.

cAMP response element-binding protein (CREB) is a key molecule in learning and memory and a core component of the molecular switch that converts short-term memory into long-term memory (Barco et al. 2003). Under normal physiological conditions in eukaryotes, target genes are mainly regulated to participate in a variety of biological functions and play a key role in regulating neuronal survival and the expression of function-related genes (Benito and Barco 2010; Sakamoto et al. 2011; Lonze and Ginty 2002), as well as synaptic formation, synaptic plasticity, and long-term memory formation (Flavell and Greenberg 2008; Frank and Greenberg 1994; Kreppel and Hart 1999). CREB must be modified and activated by autophosphorylation to regulate the transcription of target genes, which is inseparable from the phosphorylation and modification of CREB (Schmid et al. 1999). Many genes are regulated by phosphorylated CREB and are targets of phosphorylated CREB. Experiments have shown that tau is also one of the target genes regulated by CREB. The expression of CREB and the downregulation of CREB activity in the brains of patients with AD can lead to abnormal overexpression of the tau protein (Flavell and Greenberg 2008; Jin et al. 2013). Phosphorylation at Ser-133 of CREB is known to be one of the major pathways of CREB activation. Phosphorylation at Ser-133 of CREB is controlled by protein kinase A (PKA). When cAMP binds to the inactive PKA tetramer regulatory subunit, PKA is activated, and the activated PKA enters the nucleus and phosphorylates CREB at Ser-133 to activate CREB (Gonzalez and Montminy 1989). Activation of CREB through PKA can improve the transcriptional activity of CREB, in some instances up to 20 times. Disruption of CREB function in the brain can lead to neurodegeneration (Mantamadiotis et al. 2002). CREB phosphorylation plays a key role in neuroprotection. Studies have shown that phosphorylation of CREB decreases in hippocampal neurons of PS1/AAβPP transgenic mice, and phosphorylation of CREB increases with a phosphodiesterase inhibitor. Cognitive function significantly improves (Gong et al. 2004), whereas inhibition of CREB phosphorylation adversely affects spatial memory consolidation in rats (Zhang et al. 2013).

PKA is an important kinase that phosphorylates many proteins and regulates their function. It is the most common kinase that phosphorylates CREB Ser-133 and plays a critical role in the formation of learning and memory (Taylor et al. 2012). Studies have shown that the expression level of phosphorylated tau protein is regulated by various kinases, and PKA plays an important role in regulating the phosphorylation of tau protein. PKA can be phosphorylated at multiple tau-phosphorylation sites, including serine 214, 356, and 396, either alone or in collaboration with other kinases, during the progression of AD (Jensen et al. 1999; Litersky et al. 1996; Wang et al. 2007). In the absence of cAMP regulation, the PKA holoenzyme molecule is an inactive tetramer composed of a regulatory subunit and catalytic subunit and exists in the form of a passivation complex. When cAMP binds to the two regulatory subunits of inactive PKA, it separates the regulatory subunit from the catalytic subunit, and releases and activates the catalytic subunit (Taskén et al. 1997). PKA catalysed subunit activation can phosphorylate some proteins in cells, change the activity of these proteins, and affect the expression of related genes. Studies have shown that activated PKA is involved in regulating the formation of long-term memory, and the catalytic subunit of PKA phosphorylates CREB and produces a series of physiological activities (Xie et al. 2016).

In summary, to further explore the mechanism of Al-induced abnormal tau phosphorylation, PC12 cells were treated with various concentrations of maltol Al to construct an Al-induced tau hyperphosphorylation model, and the mRNA and protein expressions of miR-200a-3p, phosphorylated tau, and PKA/CREB pathways in the model were detected. A double luciferase reporting test was used to verify the target gene of miR-200a-3p, transfection miR-200a-3p inhibitor was used for cell transfection intervention, and the low expression model of miR-200a-3p was established to explore whether miR-200a-3p regulates abnormal phosphorylation of tau. This provides a new direction for studying the mechanism of Al-induced neurotoxicity.

## Materials and Methods

### Reagents

Aluminium trichloride, urethane, hydrogen peroxide (Sigma-Aldrich, St. Louis, MO, USA), maltolate (Sigma-Aldrich, St. Louis, MO, USA), anti-phospho-CREB (Ser133) (Cell Signalling, USA), anti-phospho-tau (Ser396), anti-PKA (Affinity, USA), anti-PRKACB (Merck, USA), anti-tau (Sercivebio, CN), anti-GAPDH mouse, goat anti-rabbit immunoglobulin (IgG), goat anti-mouse IgG, miRNA extraction kit, high sensitivity chemiluminescence liquid ECL, protein quantitative kit, tissue protein extraction kit, lane marker loading buffer (Reducing, 5x), EasyQuick RT MasterMix, Acry (Beijing ComWin Biotech Co., Ltd. CN), all-in-one miRNA qRT-PCR detection kit, primer for miR-200a-3p, primer for U6 (FulenGen, CN), DYC Z-24DN electrophoresis tank DYCP-40C semi-dry membrane transfer instrument (Liuyi instrument factory, Beijing), Bio-Rad chemiluminescence imaging system (Bio-Rad, USA), and ethyl carbamate (Sigma-Aldrich, St. Louis, MO, USA) were used in this study.

### Al(mal)_3_ Preparation

Milli-Q water was placed in the intercellular space under high pressure, and ultraviolet light was used. AlCl_3_·6H_2_O (0.198 g, Sigma-Aldrich, St. Louis, MO, USA) and maltol (0.3024 g) were each added to 40 mL of high pressure Milli-Q water. Maltol was shaken in a high-temperature water bath until dissolved. NaOH (0.4 g) was added to 4 mL high-pressure Milli-Q water. Al(mal)_3_ was freshly prepared for each experiment by mixing these solutions in equal volumes, adjusting the PH to 7.4 using NaOH, and filtering them through 0.22-μmol/L syringe filters.

### Cell Culture

PC12 cells were maintained in the laboratory. The cells were cultured in high-glucose Dulbecco’s modified Eagle medium (DMEM) containing 10% (v/v) foetal bovine serum (FBS) and 1% (v/v) penicillin–streptomycin at 37 °C in a humidified atmosphere of 5% CO_2_ and 95% air. Following the treatment of cells using 200 µmol/L Al(mal)_3_ for 24 h, cell viability reached 70–80% with clearly visible neurotoxic effects.

### Cell Viability

Cells from cell suspensions were counted using a microscope; the number of cells per well inoculated into the 96-well plate was approximately 1 × 10^5^. D-Hhank’s solution (100 μL) was added to each well in the outermost circle of a 96-well plate without inoculating the cells. After 24 h, the cell density was observed to be up to 50–60% using a microscope, then the culture solution was removed, changed after poisoning, and the 96-well plate was replaced into the incubator. After 24 h, the 96-well plates were removed, and the culture medium in the well was absorbed and discarded. Ten microlitres of CCK8 (MCE, USA) and 100 μL of the culture medium were added to each well. The suspensions in the 96-well plate were mixed well and replaced into the incubator for 1 h. The absorbance of each well was measured using a microplate reader. The wavelength of the microplate reader was set to 450 nm.

### Dual Luciferase Reporter Gene

The luciferase reporter (200 ng/well) and Renilla luciferase vector (10 ng/well; pRL-CMV; Hanbio, China) were transfected into HEK293T cells at 24 h post-seeding into a 24-well plate (1 × 10^5^ cells per well) using Lipofectamine TM 2000 (Han bio, CN). Luciferase activity was measured using a Dual luciferase Reporter Assay kit (Han bio, CN) at 24 h post-transfection, according to the manufacturer’s instructions.

Complete culture medium (2 mL) was added to the blank control group, 200 μmol/L Al(mal)_3_ to each well, and 1950 μL complete culture medium and 50 μL Entranster TM-R4000 (Engreen, CN) dilution were added to each well in the negative transfection group. In the NC group, 1900 μL complete medium and 100μL of the NC transfection complex (Rib. Bio, San Diego, CN, USA) were added to each well. Complete culture medium (1900 μL) and 100 μL miR-200a-3p inhibitor transfection complex (Rib. Bio, China) were added to each well of the positive transfection and positive transfection + 200 μmol/L Al(mal)_3_ groups, and the mixture was incubated for 24 h.

Solution A: 10 μL DMEM + 0.16 μg R-PrkACB-3′UTR target plasmid + 5 pmol rNO-miR-200a-3p/negative control (NC). Solution B: 10μL of DMEM + 0.3 μL transfection reagent. They were then mixed into the transfection mixture and incubated at room temperature for 20 min. The 96-well plates for culturing 293 T cells were removed and the fluid in the well was discarded. The transfection mixture was added, cultured for 6 h, and incubated and transfected for 48 h after the fluid was changed. The cells were collected for fluorescence detection. Lysis buffer (100μL) was added into the 96-well plate to complete the cell lysis and centrifuged at 12000 rpm at 4 °C for 10 min. The supernatant was added to 100μL of luciferase reaction reagent (Han Bio, CN) for the enzyme-plate assay to obtain the intensity of firefly luciferase (F-LUC), which was the internal reference value. Then, 100μL of luciferase reaction reagent II was added for detection in a microplate analyser to obtain the R-LUC, which was used to report the gene luminescence value (relative fluorescence value = F-LUC/R-LUC).

### Reverse Transcription Quantitative Polymerase Chain Reaction (RT-qPCR)

Total RNA from the serum samples was extracted using TRIzol^®^reagent (CoWin Biotech, CN), quantified using a NanoDrop spectrophotometer, and treated with RNAase-free DNase I (Invitrogen) as recommended by the manufacturer. For miRNA expression analysis, 0.5 μg RNA was added to a polyA tail using the miDETECT A Track™ miRNA qRT-PCR Starter kit (RiboBio, Guangzhou, China), and reverse transcribed with miDETECT A Track™ Uni-RT primer. Specific primers and SYBR Green mix used to detect miRNAs were purchased from RiboBio Co., Ltd. The U6 endogenous control was used for normalisation. For mRNA expression analysis, 0.5 μg RNA was reverse transcribed using a Superscript RT reagent kit (Takara). The specific primers used for real-time PCR are listed in Table 1. The PCR conditions were as follows: pre-denaturation at 95 °C for 10 min, followed by 40 cycles of denaturation at 95 °C for 10 s, annealing at 60 °C for 20 s, and extension at 72 °C for 10 s. GAPDH was used as an internal standard for mRNA quantification. Melting curve analysis was used to determine primer specificity.

**Table 1:** Primer sequences used for reverse transcription-quantitative PCR

Gene	Forward primer (5′–3′)	Reverse primer (5′–3′)
miR-200a-3p	TAACACTGTCTGGTAAGGATGTAA	GGCCAACCGCGAGAAGATG
U6	CTCGCTTCGGCAGCACA	AACGCTTCACGAATTTGCGT
mRNA-PRKACB	GTCACTCCTGCTTACGGACT	ATTACTCGGGGGAGGGTTCT
mRNA-GAPDH	AGTGCCAGCCTCGTCTCATA	TGAACTTGCCGTGGGTAGAG

### Western Blotting Analysis

The samples were loaded and separated on 7% sodium dodecyl sulphate–polyacrylamide gel electrophoresis (SDS-PAGE) to detect PRKACB, PKA, CREB, and tau. Proteins were transferred to PVDF membranes using a semi-dry transfer unit (1.5 mA/cm^2^; Bio-Rad, Hercules, CA, USA). The membranes were saturated and blocked with 5% fat-free milk incubated overnight at 4 °C in rabbit anti-PRKACB (1:2000) (Merck, USA), anti-PKA (1:1000), anti-p-tau (Ser396) (1:1000) (Affinity, USA), anti-tau (1:500) (Servicebio, CN), anti-p-CREB (Ser133) (1:2000) (Cell Signaling, USA), and mouse anti-GAPDH (1:3000; ComWin Biotech Co., Ltd., Beijing). Blots were washed thrice for 10 min in TBST at room temperature and then incubated for 2 h at 37 °C with one of the following antibodies: anti-rabbit IgG (1:3000; Beijing ComWin Biotech Co., Ltd.) or anti-mouse IgG (1:3000, Beijing ComWin Biotech Co., Ltd.). After washing thrice for 10 min in TBST, immuno-labelled protein bands were detected using an ECL western blot detection kit (CoWin Biotech, CN). Images were obtained using a chemiluminescence imaging system (Bio-Rad). Densitometric quantification of the immunoblots was performed using the Quantity One software (Bio-Rad).

### Statistical Analysis

All data are expressed as means ± SD. Data were analysed using by a paired *t*-test for two-group comparisons and one-way analysis of variance (ANOVA) for comparison of more than two groups using the SPSS statistical software package, Version 17.0 (SPSS Inc., Chicago, IL). Statistical significance was set at *P* value < 0.05.

## Results

### Maltol Al Exposure Group with Various Concentrations

#### Effects of Maltol Al on Morphology of PC12 Cells

**Fig. 1:**
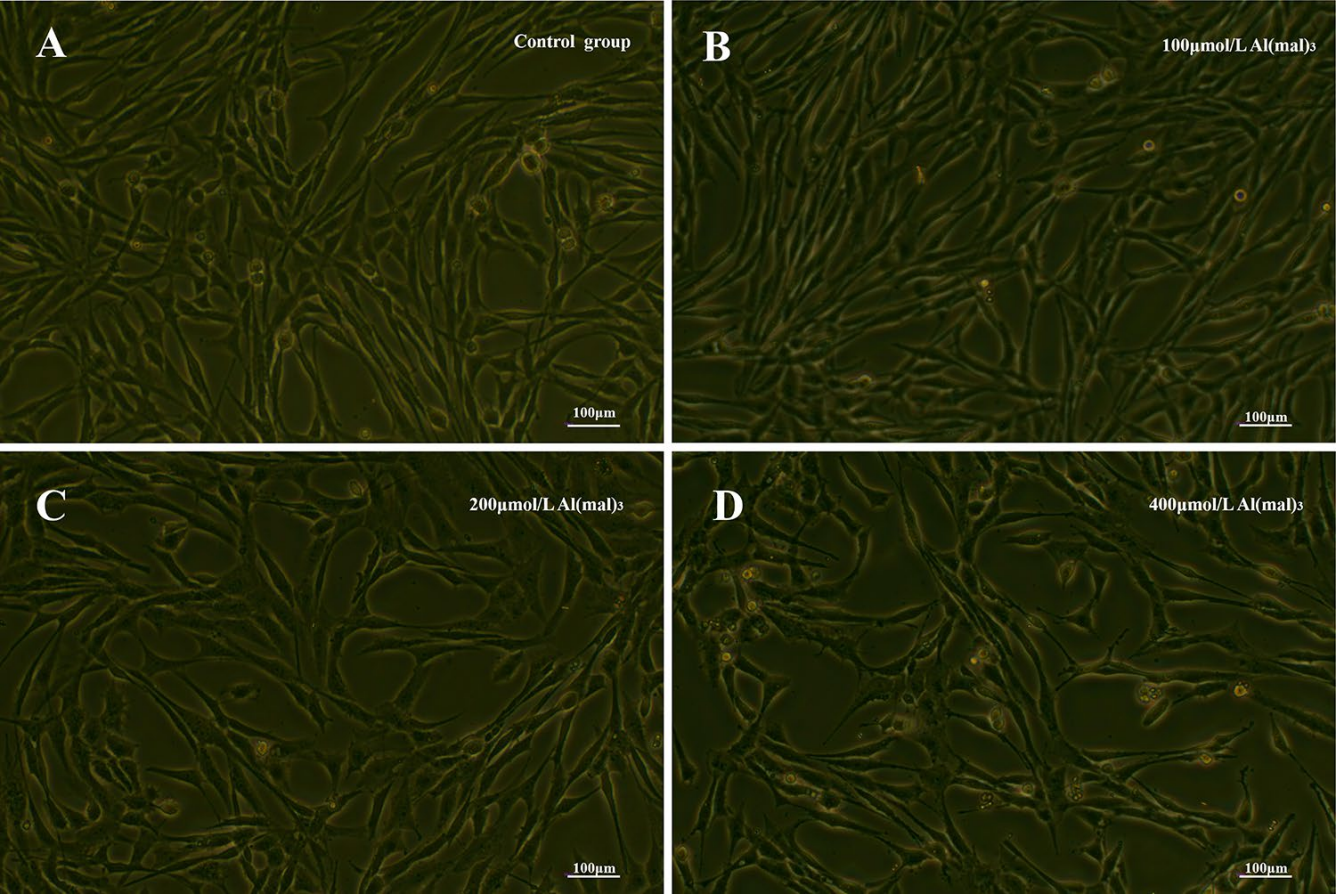
Cell morphology of groups exposed to various concentrations of maltol Al (200 ×). Control group (**A**), 100 μmol/L Al (mal)_3_ group (**B**), 200 μmol/L Al(mal)_3_ group (**C**), and 400 μmol/L Al(mal)_3_ group (**D**)

As shown in Fig. 1, under normal conditions, the cell body is fusiform, with clear boundaries, tight intercellular connections, and vigorous growth. The morphology of PC12 cells treated with various concentrations of maltol Al for 24 h was observed using a microscope. The cells in the low-dose group were slightly reduced compared with the normal control group, but the reduction was not clearly visible (Fig. 1B). In the medium-dose group, cell density decreased and connections between cells decreased (Fig. 1C), whereas in the high-dose group, the cell body contracted, the intercellular connections were sparse, the dead cells increased, and the cell density decreased significantly (Fig. 1D).

#### Cell Viability of PC12 of Maltol Al Treatment

**Fig. 2:**
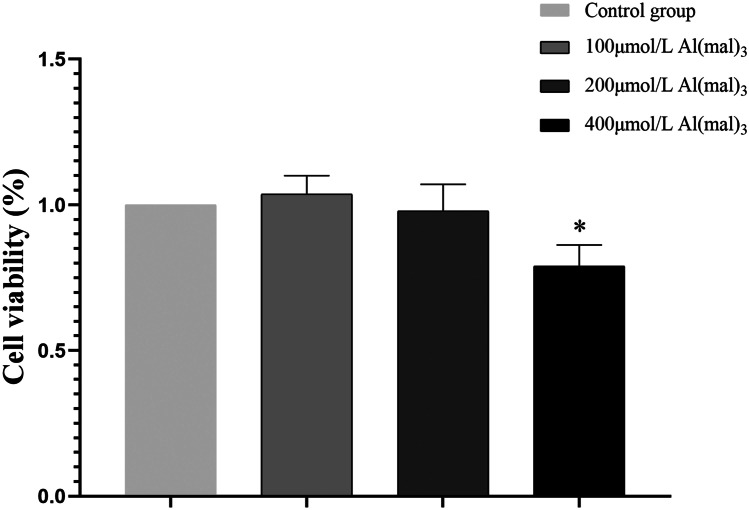
Cell viability of groups exposed to various concentrations of maltol Al. Values are represented as mean ± SD compared with the control group. *P < 0.05

PC12 cells were exposed to maltol Al at various concentrations for 24 h, and cell viability was determined using the CCK8 kit. As shown in Fig. 2, the cell viability of the low-dose group was slightly reduced compared with the control group under normal conditions. The cell viability of medium-dose group was not significantly decreased. The cell viability of the high-dose group was significantly decreased with statistical significance (*P* < 0.05).

#### Effects of Maltol Al on miR-200a-3p Expression in PC12 Cells

**Fig. 3:**
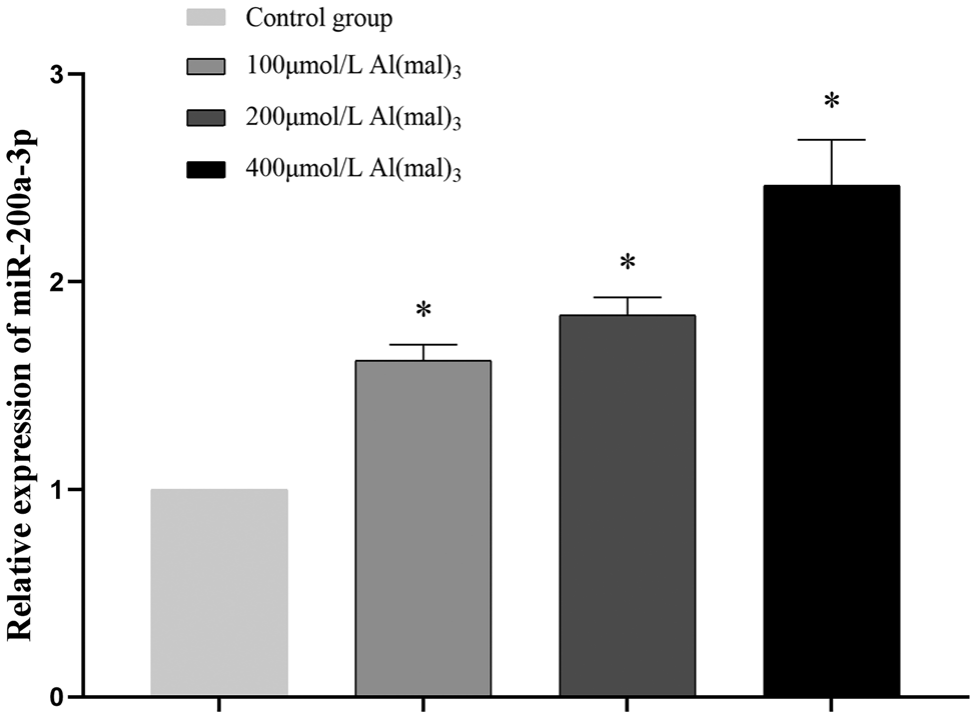
Expression levels of miR-200a-3p in groups exposed to various concentrations of maltol Al. Values are represented as mean ± SD compared with the control group. *P < 0.05

After PC12 cells were exposed to maltol Al at various concentrations for 24 h, the expression of miR-200a-3p was detected using RT-PCR. Compared with the control group, the expression of miR-200a-3p in 100 μmol/L Al(mal)_3_ group increased by 0.62 times, that of the 200 μmol/L Al(mal)_3_ group increased by 0.84 times, and that of the 400 μmol/L Al(mal)_3_ group increased by 1.47 times. With an increase in treatment concentration, the expression level in miR-200a-3p gradually increased, and the increase of miR-200a-3p expression level among the exposed groups was statistically significant (*P *< 0.05), as shown in Fig. 3.

#### Effects of Maltol Al on mRNA-PRKACB and PRKACB Protein Expression

**Fig. 4:**
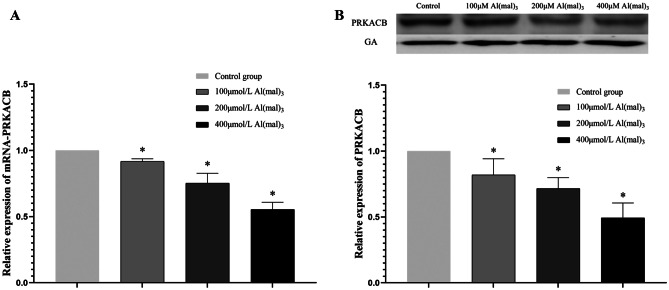
mRNA-PRKACB and PRKACB protein expression. Values are represented as mean ± SD compared with the control group. *P < 0.05

After PC12 cells were exposed to maltol Al at various concentrations for 24 h, RT-PCR was used to detect the relative expression of RNA. GA was used as an internal reference to calculate the 2^−ΔΔCT^ value and analyse the relative expression level of mRNA-PRKACB in PC12 cells. Compared with the control group, the mRNA-PRKACB expression in the 100 μmol/L Al(mal)_3_ group decreased by 8.3%, and that in the 200 μmol/L Al(mal)_3_ group decreased by 24.7%. The mRNA-PRKACB expression in the 400 μmol/L Al(mal)_3_ group was decreased by 44.6%. With increasing Al(mal)_3_ concentration, the expression level of mRNA-PRKACB gradually decreased in the exposed groups (*P *< 0.05), as shown in Fig. 4A.

Western blotting was used to detect PRKACB protein, and Fiji software was used to analyse the grey value of the protein bands and relative protein expression. Compared with the control group, the protein expression of PRKACB decreased by 18.1% in the low-dose, by 28.4% in the medium-dose, and by 44.6% in the high-dose groups. The expression of PRKACB decreased with increasing exposure concentration (*P *< 0.05), and the expression of PRKACB protein in each group decreased gradually (*P *< 0.05), as shown in Fig. 4B.

#### Effects of Maltol Al on the Expression of PKA, P-CREB (Ser133), and p-tau (Ser396) in PC12 Cells

PKA, P-CREB (Ser133), and p-tau (Ser396) proteins were detected using western blot after 24 h of Al exposure in PC12 cells. The grey values of the protein bands were analysed using the Fiji software. The relative expression levels of PKA, p-CREB (Ser133), and p-tau (Ser396) were analysed.

**Fig. 5:**
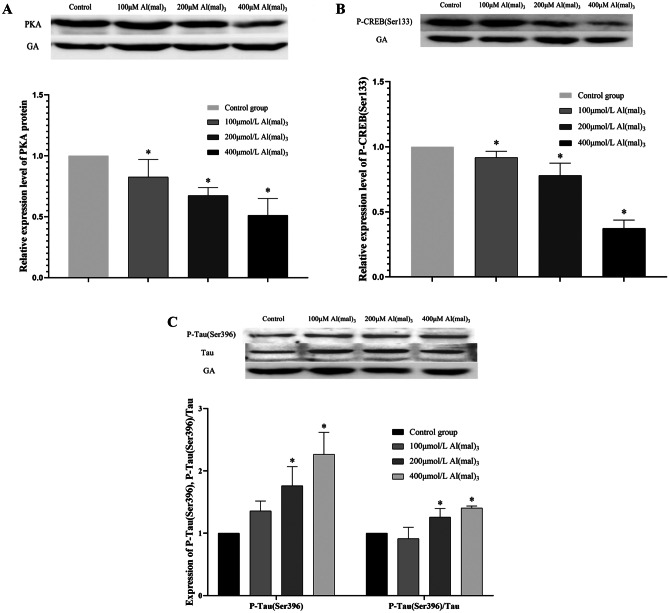
The relative expression of p-tau (Ser396) protein in groups exposed to various concentrations of maltol Al. Values are represented as mean ± SD compared with the control group. *P < 0.05

Compared to the control group, PKA protein expression in the 100, 200, and 400 μmol/L Al(mal)_3_ groups decreased by 17.5%, 32.6%, and 48.8% (*P *< 0.05), respectively, as shown in Fig. 5A.

Compared with the control group, the expression of p-CREB (Ser133) protein in the 100 μmol/L Al(mal)_3_ group decreased, but there was no statistical significance (*P* > 0.05). The relative expression of p-CREB (Ser133) in the 200 and 400 μmol/L Al(mal)_3_ groups decreased by 22.0% and 62.7% (*P *< 0.05), respectively, as shown in Fig. 5B.

Compared to the control group, the expression of p-tau (Ser396) protein in the 100 μmol/L Al(mal)_3_ group was not significantly increased (*P* > 0.05), whereas the expression of p-tau (Ser396) protein in the 200 and 400 μmol/L Al(mal)_3_ groups increased 0.76 times and 1.27 times (*P *< 0.05), respectively. Compared to the control group, the change in the p-tau (Ser396)/tau ratio in the 100 μmol/L Al(mal)_3_ group was not statistically significant (*P* > 0.05), and the ratio of p-tau (Ser396)/tau in the 200 and 400 μmol/L Al(mal)_3_ groups increased (*P *< 0.05), as shown in Fig. 5C.

### Dual Luciferase Reporter Assay

**Fig. 6:**
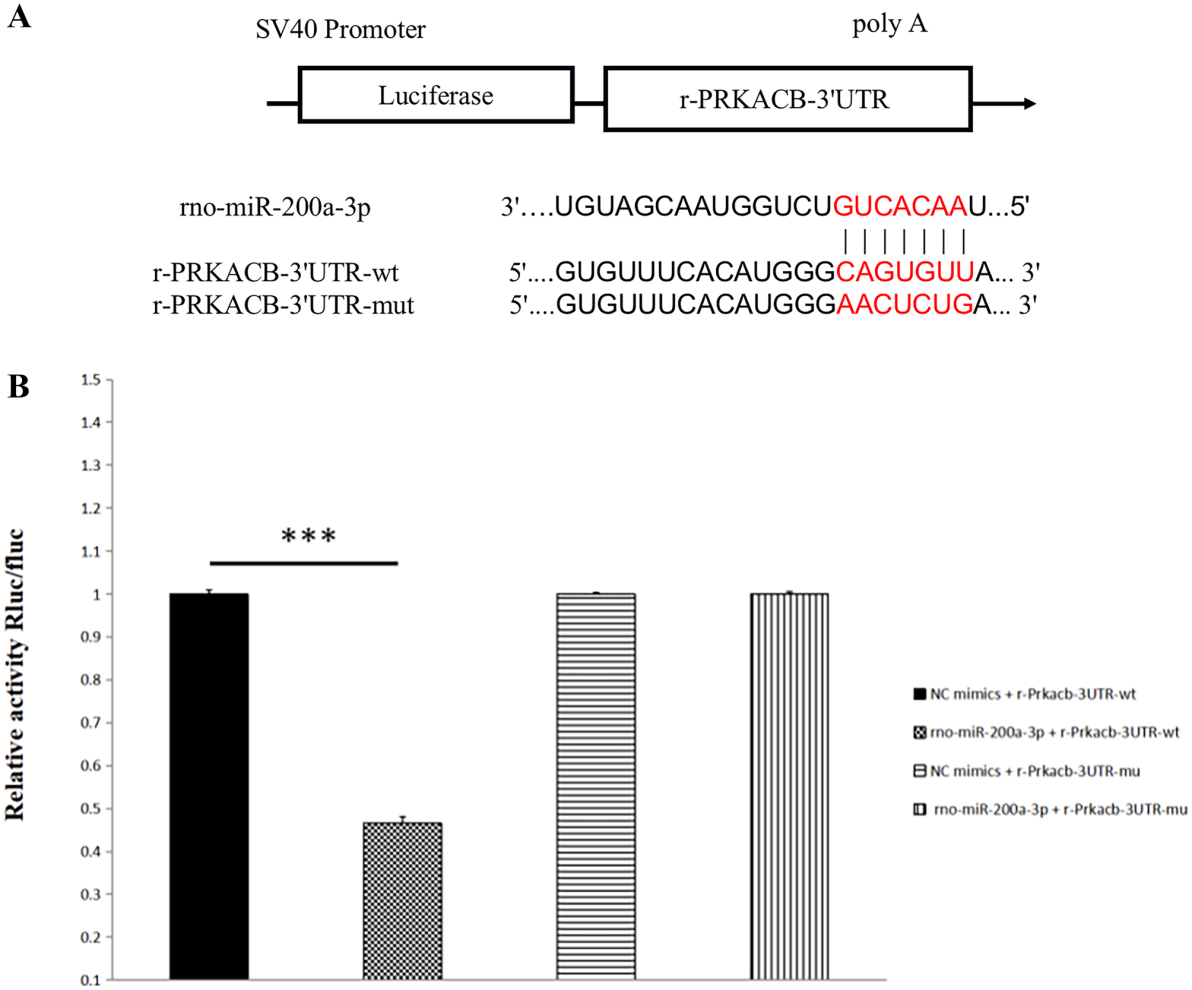
**A** Schematic diagram of miR-200a-3p binding to PRKACB-3′-UTR target sites. **B** miR-200a-3p targeting PRKACB was determined using a dual luciferase assay. Values are represented as mean ± SD, compared with the control group. *P < 0.05

The miRNA target prediction database TargetScan was used to identify the binding site of miR-200a-3p and PRKACB, which showed that miR-200a-3p and mRNA-PRKACB had a clear 3′-UTR target, as shown in Fig. 6A.

The predicted target was verified using a double luciferase assay. The 293 T cells were transfected with luciferase plasmids wt-PRKACB/mut-PRKACB and miR-200a-3p/NC mimics to detect luciferase activity and, to verify the targeted binding of miR-200a-3p to PRKACB.

The dual-luciferase reporter assay results showed that miR-200a-3p decreased luciferase activity in the wt-PRKACB vector with statistical significance (*P *< 0.05) and, did not change the luciferase activity of mut-PRKACB (*P* > 0.05), indicating that miR-200a-3p can specifically bind to PRKACB, as shown in Fig. 6B.

#### miR-200a-3p Inhibitor Transfection

##### Effects of miR-200a-3p Inhibition on PC12 Cell Morphology

**Fig. 7:**
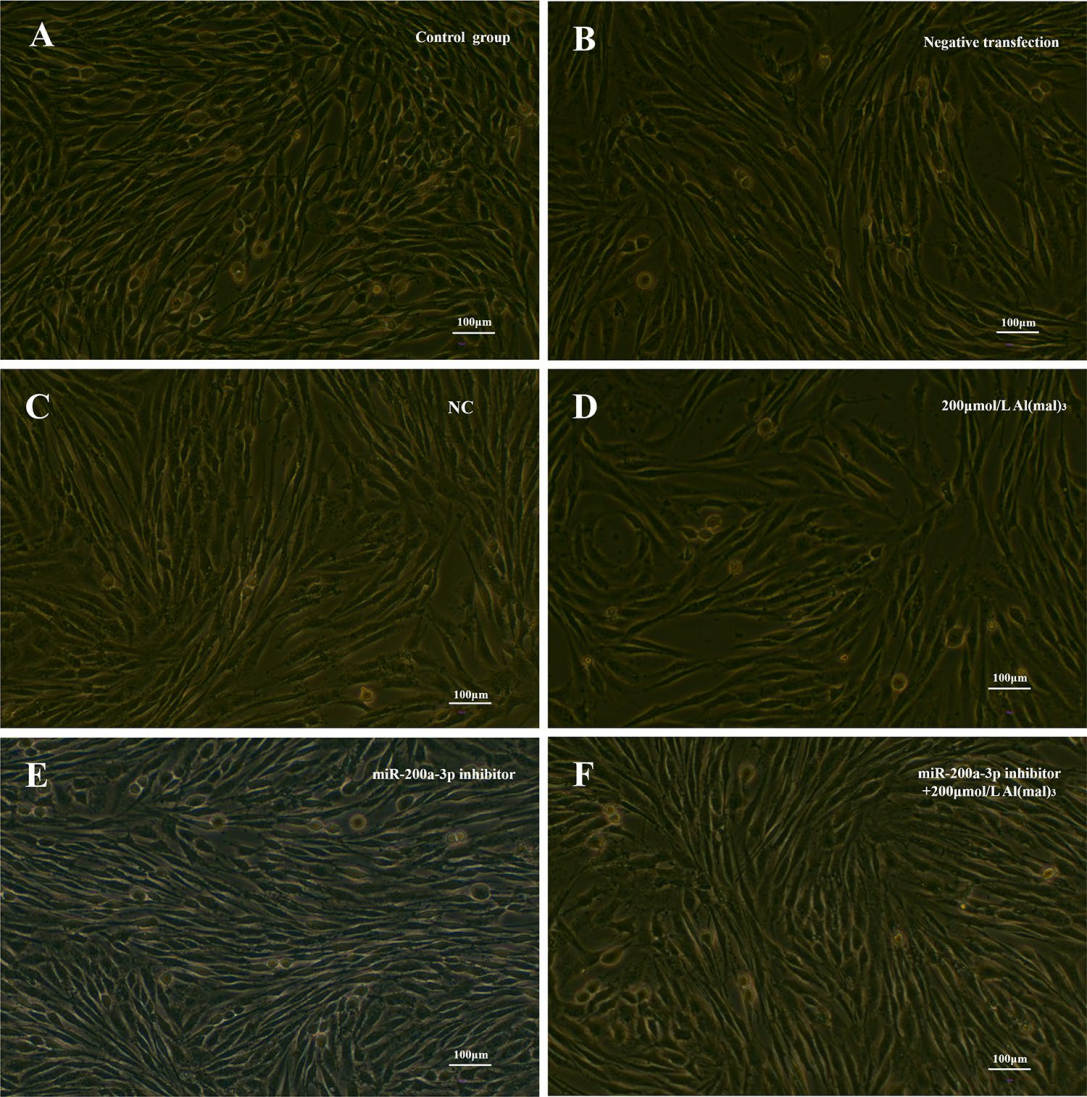
Cell morphology of PC12 cells after miR-200a-3p inhibition. In the control group, the cell morphology was normal, cell numbers were large, and good growth was observed (**A**). The cell morphology of the negative transfection group (**B**) and non-specific control group (**C**) was similar to that of the control group. In 200 μM Al(mal)_3_ Al exposure group, cell death increased to some extent and intercellular connectivity decreased (**D**). In the miR-200a-3p inhibitor group, cell numbers increased and intercellular junctions increased (**E**). After transfection with the miR-200a-3p inhibitor, the 200 μM Al(mal)_3_ Al group was exposed and the cell morphology was normal, similar to that of the control group (**F**)

After transfection with the miR-200a-3p inhibitor, cell morphology was observed using a microscope 24 h after Al exposure. Compared to the control group, maltol Al exposure increased cell death and reduced intercellular connectivity in the test groups. After transfection with miR-200a-3p inhibitor, compared with the 200 μM Al(mal)_3_ group, the number of cells and cell connections increased, as shown in Fig. 7.

##### Effect of miR-200a-3p Inhibition on miR-200a-3p Expression in PC12 Cells

**Fig. 8:**
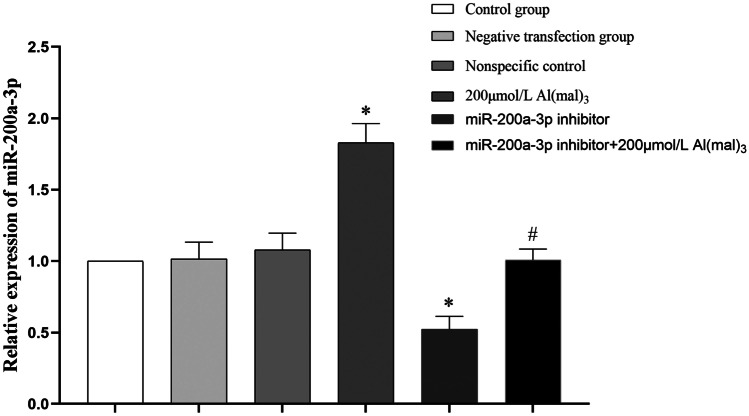
Expression of miR-200a-3p in PC12 cells after miR-200a-3p inhibition. Values are represented as mean ± SD, compared with the control group, *P < 0.05, and compared with the 200 μmol/L Al(mal)_3_ group, #*P* < 0.05

Maltol Al treatment was performed after transfection with the miR-200a-3p inhibitor, and PC12 cells were analysed after exposure to the samples. RNA expression was detected using RT-PCR, and the relative expression of miR-200a-3p was obtained by calculating 2^−ΔΔCT^ with U6 as an internal reference. Compared to the control group, there was no significant difference in miR-200a-3p expression between the negative transfection and NC groups (*P* > 0.05). miR-200a-3p expression in the positive transfection group decreased by 47.7% (*P *< 0.05). Compared with the 200 μmol/L Al(mal)_3_ group, miR-200a-3p expression in the transfection + 200 μmol/L Al(mal)_3_ group decreased by 44.9% (*P* < 0.05), as shown in Fig. 8.

##### Effects of miR-200a-3p Inhibition on mRNA-PRKACB and PRKACB Protein Expression in PC12 Cells

**Fig. 9:**
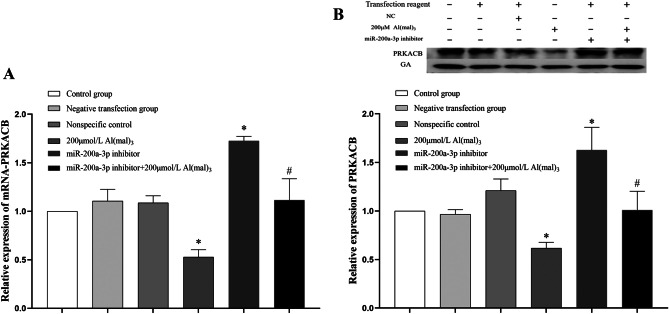
Relative expression levels of mRNA-PRKACB and PRKACB protein in PC12 cells. Values are represented as mean ± SD, compared with the control group, *P < 0.05, and compared with the 200 μmol/L Al(mal)_3_ group, #*P* < 0.05

PC12 cells were sampled 24 h after transfection with the miR-200a-3p inhibitor. The relative expression of RNA was detected using RT-PCR and GA was used as an internal reference to calculate the 2^−ΔΔCT^ value for analysis. Compared with the control group, there was no significant difference in the expression of mRNA-PRKACB in the negative transfection and NC groups (*P* > 0.05). mRNA-PRKACB expression in the positive transfection group increased 0.73 times (*P *< 0.05). Compared with the 200 μmol/L Al(mal)_3_ group, the mRNA expression of PRKACB in the positive transfection + 200 μmol/L Al(mal)_3_ group increased 1.11 times (*P *< 0.05), as shown in Fig. 9A.

Western blotting was used to detect protein expression. Fiji software was used to analyse the protein bands, and the relative expression levels of the tested proteins were calculated using the grey value. Compared with the control group, there was no significant difference in PRKACB protein expression between the negative transfection and NC groups (*P* > 0.05), and the expression of PRKACB protein in the positive transfection group was increased 0.73 times (*P *< 0.05). Compared with the 200 μmol/L Al(mal)_3_ group, the expression of PRKACB protein in the positive transfection + 200 μmol/L Al(mal)_3_ group was increased 0.63 times (*P *< 0.05), as shown in Fig. 9B.

##### Effects of miR-200a-3p Inhibition on Protein Expression of PKA, P-CREB (Ser133), and p-tau (Ser396) in PC12 Cells

**Fig. 10:**
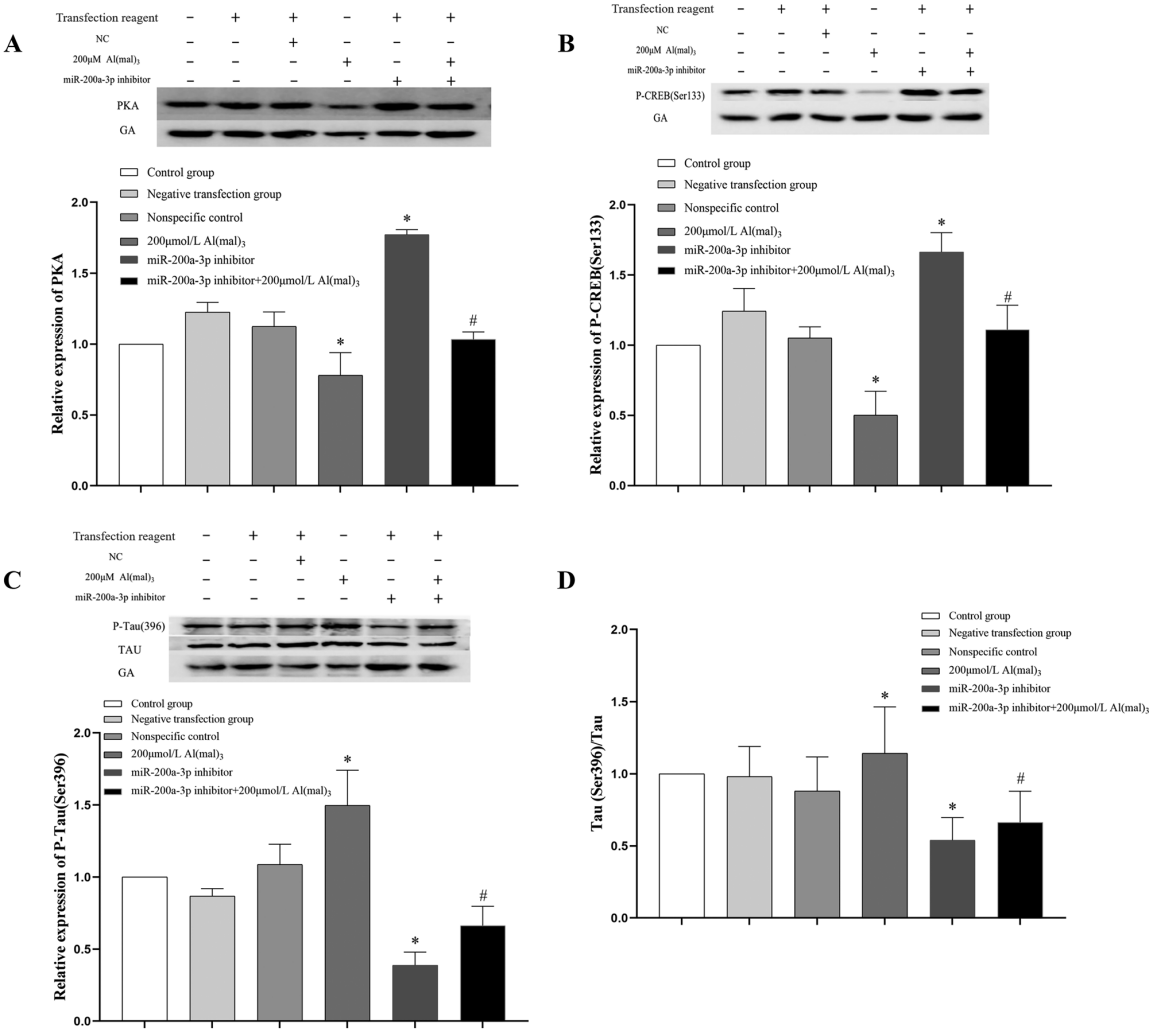
The expression levels of PKA, P-CREB (Ser133), and p-tau (Ser396) in PC12 cells were inhibited by miR-200a-3p. Values are represented as mean ± SD, compared with the control group, *P < 0.05, and compared with the 200 μmol/L Al(mal)_3_ group, #*P* < 0.05

Twenty-four hours after transfection with miR-200a-3p inhibitor, PC12 cells were sampled and protein bands were analysed using western blotting and Fiji software. The relative expression levels of PKA, p-CREB (Ser133), and p-tau (Ser396) were calculated using grey values. Compared with the control group, there was no significant difference in PKA protein expression between the negative transfection and NC groups (*P* > 0.05). PKA protein expression in the positive transfection group increased 0.77 times (*P *< 0.05). PKA protein expression in the positive transfection + 200 μmol/L Al(mal)_3_ group was 0.33 times higher than that in the 200 μmol/L Al(mal)_3_ group (*P *< 0.05), as shown in Fig. 10A.

Compared with the control group, there was no significant difference in p-CREB (Ser133) protein expression between the negative transfection and NC groups (*P* > 0.05). The protein expression of P-CREB (Ser133) in the positive transfection group increased 0.66 times (*P *< 0.05). PKA protein expression in the positive transfection + 200 μmol/L Al(mal)_3_ group was 1.21 times higher than that in the 200 μmol/L Al(mal)_3_ group (*P *< 0.05), as shown in Fig. 10B.

Compared to the control group, there was no significant difference in the expression of p-tau (Ser396) protein between the negative transfection and NC groups (*P* > 0.05). The expression of p-tau (Ser396) protein in the positive transfection group decreased 61.1% (*P *< 0.05). The expression of p-tau (Ser396) protein in the positive transfection + 200 μmol/L Al(mal)_3_ group was reduced by 55.2% compared with the 200 μmol/L Al(mal)_3_ group (*P *< 0.05), as shown in Fig. 10C.

Compared with the control group, there was no significant difference in the ratio of p-tau (Ser396)/tau protein between the negative transfection and NC groups (*P* > 0.05), whereas the p-tau (Ser396)/tau ratio in the positive transfection group decreased (*P *< 0.05). In the positive transfection + 200 μmol/L Al(mal)_3_ group compared with the 200 μmol/L Al(mal)_3_ group, p-tau (Ser396)/tau ratio decreased (*P *< 0.05), as shown in Fig. 10D.

## Discussion

Al is the most abundant metal in the Earth crust. Several studies have shown that the damaging effect of Al on the nervous system is often reflected through its close correlation with various neurodegenerative diseases, such as AD, amyotrophic lateral sclerosis, and dialysis encephalopathy (Kandimalla et al. 2016a). Studies have shown that long-term exposure to Al can cause accumulation in the body. Al can accumulate in the brain through the blood–brain barrier and placental barriers, leading to a variety of neurodegenerative diseases (Ferreira et al. 2008). Studies have shown that Al can cause neurotoxicity and plays an important role in the occurrence and development of AD (Kandimalla et al. 2016b; Bondy 2016).

Al is too chemically reactive to exist as a free metal in nature. In this study, maltol was used as a coupling agent to combine with Al to generate maltol Al that infects cells. Maltol is a by-product of sucrose hydrolysis or starch thermal degradation, which is less harmful to the human body and is often used as a food additive in the food processing industry. Maltol and Al can be combined to produce electrically neutral maltol Al, which is not easily degraded, has a stable structure, and is not easily hydrolysed at room temperature. Under normal physiological pH, maltol Al can release a large number of Al ions, which makes it highly bioavailable; it can thus be absorbed and accumulated in the brain tissue, which is suitable for relevant studies on neurotoxicity (Vasudevaraju et al. 2008). The PC12 cell line is a common nerve cell line derived from adrenal pheochromocytoma of *Rattus norvegicus*, which is a sympathetic nervous system tumour cell with a stable growth state that is often used in gene transfection studies (Li et al. 2021b). Therefore, maltol Al was selected as the dye venom to infect the PC12 cells with for subsequent experimental detection.

AD has two iconic pathological features, namely senile plaques and neurofibrillary tangles, which are mainly formed by hyperphosphorylation of tau protein (Torreilles and Touchon 2002). Studies have shown that hyperphosphorylation of tau protein plays a major role in the neuropathology of AD (Hampel et al. 2010). In patients with AD, the earliest damage occurs in the tangles of cerebral endodermis cells, and the formation of the tangles involves the interaction between Al and the highly phosphorylated tau protein (Walton 2010). Other studies have shown that Al is closely related to the abnormal phosphorylation of tau protein, and Al can cause the pathological reaction thereof (Walton 2007, 2006). In this study, to verify whether Al can induce tau hyperphosphorylation, maltol Al was exposed to PC12 cells, and phosphorylated tau protein was detected. The results showed that the expression of phosphorylated tau protein changed with an increase in maltol Al exposure concentration, and the expression of phosphorylated tau protein increased significantly in the 200 and 400 μmol/L Al(mal)_3_ groups (*P *< 0.05). In addition, Buee et al. (2000) found that abnormal hyperphosphorylation of tau protein is one of the characteristics of adult neurodegenerative diseases (such as AD and PD). The results of our study are consistent with these findings; therefore, we propose that Al can lead to hyperphosphorylation of the tau protein.

CREB is the core component of the molecular switch that converts short-term memory into long-term memory and plays a key role in learning and memory (Barco et al. 2003). CREB is a ubiquitous nuclear transcription factor that can regulate the transcription of target genes, and it must be modified and activated by autophosphorylation to play its role in regulating the transcription of target genes (Schmid et al. 1999). Studies have shown that the PKA/CREB pathway plays an important role in memory (Kandel 2012). The tau gene is one of the target genes regulated by CREB, and downregulation of the PKA/CREB signalling pathway can lead to learning and memory defects in patients with AD and in mice (Puzzo et al. 2005; Yamamoto-Sasaki et al. 1999; Matsuzaki et al. 2006; Liang et al. 2007). In the brains of patients with AD, downregulation of CREB expression or activity can lead to overexpression of phosphorylated tau protein (Flavell and Greenberg 2008; Jin et al. 2013). Phosphorylation at Ser-133 is one of the main pathways involved in CREB activation. Phosphorylation at this site is controlled by PKA; PKA can promote the phosphorylation of CREB, and the transcriptional activity of CREB increases. Destruction of CREB function in the brain can lead to neurodegeneration, and CREB phosphorylation plays an important role in neuroprotection.

In this study, a maltol Al exposure model was constructed to detect the protein expression levels of PKA, p-CREB (Ser133), and p-tau (Ser396). The results showed that with an increase in maltol Al exposure concentration, the protein expression levels of PKA decreased gradually (*P < *0.05), the protein expression of P-CREB (Ser133) decreased (*P < *0.05), and the expression of p-tau (Ser396) protein increased (*P < *0.05). The results indicated that maltol Al exposure can inhibit the activity of the PKA/CREB signalling pathway, suggesting that the inhibition of PKA/CREB signalling pathway activity may be an upstream regulatory pathway leading to tau hyperphosphorylation. The detection results of the miR-200a-3p low expression model showed that compared with the control group, the protein expression levels of PKA and P-CREB (Ser133) increased (*P <* 0.05), and the expression of p-tau (Ser396) protein decreased (*P <* 0.05), compared with the 200 μmol/L Al(mal)_3_ group. The protein expression levels of PKA and P-CREB (Ser133) in the positive transfection + 200 μmol/L Al(mal)_3_ group increased (*P <* 0.05) and the expression of p-tau (Ser396) protein decreased (*P <* 0.05), which is consistent with previous studies that the transcriptional activity of CREB is reduced, and the destruction of CREB function in the brain can lead to the expression of the protein of neurodegeneration (Mantamadiotis et al. 2002). These results indicate that Al may lead to a decrease in the activity of the PKA/CREB pathway and an increase in the phosphoryp-tau (Ser396) protein decreasedlation level of the tau protein.

miRNAs have gene regulatory functions and can negatively regulate target genes by pairing with the 3′-UTR of target messenger genes (Rajasekaran et al. 2015; Kinose et al. 2014). miRNAs extensively regulate multiple aspects of the nervous system by participating in post-transcriptional regulation of biological gene expression. Studies have shown that miRNAs are involved in various neurodegenerative diseases. For example, in the pathological processes of AD and PD (Ebert and Sharp 2012), miRNA may be potential regulatory factors for AD-related target genes (Liu et al. 2014). miR-200a-3p belongs to the miR-200 family, which regulates cell differentiation, apoptosis, and proliferation and plays an important role in human diseases such as cancer and AD (Feng et al. 2014; Wu et al. 2017). Many studies have shown that miR-200a-3p plays an important role in AD, but there are variations in miR-200a-3p levels detected in various AD models. Some studies have shown that miR-200a-3p expression is downregulated during AD (Liu et al. 2014). However, other studies have shown that the expression of miR-200a-3p was increased in the hippocampus of patients with AD and APP/PS1 mice (Zhang et al. 2017; Lau et al. 2013). The model was constructed to elucidate whether the expression level of miR-200a-3p in Al-exposed PC12 cells changed and whether the change in miR-200a-3p expression level affected the change in tau phosphorylation level. PC12 cells were exposed to various concentrations of maltol Al to construct the tau hyperphosphorylation model, and then miR-200a-3p was detected. The results showed that the expression level of miR-200a-3p increased with increasing maltol Al exposure.

To verify the relationship between miR-200a-3p levels and Al exposure, the miR-200a-3p low expression model was constructed by inhibiting the expression of miR-200a-3p in PC12 cells. RT-PCR results showed that the expression of miR-200a-3p in the positive transfection group was lower than that in the control group (*P *< 0.05). Compared with the 200 μmol/L Al(mal)_3_ group, miR-200a-3p expression in the positive transfection + 200 μmol/L Al(mal)_3_ group was decreased (*P *< 0.05), indicating that the low expression model of miR-200a-3p in PC12 cells was successfully constructed. Western blot analysis showed that the expression of p-tau (Ser396) protein was decreased compared with that of the control group (*P *˂ 0.05). Compared with the 200 μmol/L Al(mal)_3_ group, the expression of p-tau (Ser396) protein in the positive transfection + 200 μmol/L Al(mal)_3_ group was decreased (*P *< 0.05). Therefore, there was an interaction between the inhibition of miR-200a-3p and the expression of tau phosphorylated by 200 μmol/L Al(mal)_3_. These results indicate that miR-200a-3p is involved in regulating the mechanism of Al induced tau phosphorylation; inhibition of miR-200a-3p expression results in decreased expression of phosphorylated tau protein.

Phosphorylation of CREB at Ser-133 is controlled by PKA. When cAMP binds to the inactive PKA tetramer regulatory subunit to activate PKA, the activated PKA enters the nucleus, where it activates CREB by phosphorylating the CREB Ser-133 site (Gonzalez and Montminy 1989). CAMP binds to the two regulatory subunits of the inactive PKA tetramer, which separates the regulatory subunit from the catalytic subunit, and releases and activates the catalytic subunit (Taskén et al. 1997). The catalytic subunit of PKA can further act on CREB, resulting in its phosphorylation to produce a series of physiological activities.

To verify whether miR-200a-3p regulates the PKA/CREB pathway by targeting PRKACB, we analysed the constructed tau protein hyperphosphorylation model and miR-200a-3p low expression model. Analysis of the detection results of the tau hyperphosphorylation model showed that with the increase in maltol Al exposure concentration, the expression of miR-200a-3p increased (*P *< 0.05), mRNA-PRKACB and PRKACB protein expression decreased (*P *< 0.05), and the protein expression of P-CREB (Ser133) in the 200 and 400 μmol/L Al(mal)_3_ groups was significantly decreased (*P *< 0.05), compared with the control group. This suggests that Al targeted PRKACB to reduce its expression by increasing miR-200a-3p expression, and then participated in reducing PKA/CREB signalling pathway activity. To verify the hypothesis, the miR-200a-3p inhibition model was tested, and it was found that, compared with the control group, the expression of miR-200a-3p in the positive transfection group was decreased (*P *< 0.05); compared with the 200 μmol/L Al(mal)_3_ group, miR-200a-3p expression in the positive transfection + 200 μmol/L Al(mal)_3_ group was decreased (*P *< 0.05); mRNA-PRKACB, PRKACB, PKA, and p-CREB (Ser133) protein expression increased (*P *< 0.05), compared with the 200 μmol/L Al(mal)_3_ group; and mRNA expression levels of PRKACB, PRKACB, PKA, and P-CREB (Ser133) in the positive transfection + 200 μmol/L Al(mal)_3_ group were increased (*P *< 0.05). These results showed that there was an interaction between the inhibition of miR-200a-3p and the expression of PRKACB, PKA, and CREB of the 200 μmol/L Al(mal)_3_ group. Therefore, Al may be involved in reducing the activity of the PKA/CREB signalling pathway by targeting PRKACB expression through increasing miR-200a-3p.

## Conclusions

Based on the data from the present study, Al exposure can increase the expression level of miR-200a-3p and target the expression of PRKACB, then participate in the reduction of PKA/CREB signalling pathway activity leading to tau hyperphosphorylation. This study provides new insight into the mechanism of Al-induced neurotoxicity.

## Data Availability

Data and materials are available from the corresponding author upon reasonable request.

## Abbreviations

miRNAs: microRNAs
CREB: cAMP response element-binding protein
PKA: protein kinase A
PRKACB: cAMP-dependent protein kinase catalytic subunit beta
AD: Alzheimer’'s disease
PD: Parkinson’'s disease
NFT: nerve fibre twineing
ncRNAs: non-coding RNAs
3′UTR3′: untranslated region
IgG: Goat anti-rabbit immunoglobulin
DMEM: Dulbecco’s modified Eagle medium
FBS: Foetal bovine serum
ANOVA: one-way analysis of variance

## References

[CR1] Alfrey A, LeGendre G, Kaehny W (1976). The dialysis encephalopathy syndrome. Possible Aluminum Intoxication, the New England Journal of Medicine.

[CR2] Barco A, Pittenger C, Kandel E (2003). CREB, memory enhancement and the treatment of memory disorders: promises, pitfalls and prospects. Expert Opin Ther Targets.

[CR3] Banks S, Pierce M, Soukup G (2020). Sensational microRNAs: neurosensory roles of the microRNA-183 family. Mol Neurobiol.

[CR4] Basak I, Patil K, Alves G, Larsen J, Møller S (2016). microRNAs as neuroregulators, biomarkers and therapeutic agents in neurodegenerative diseases. Cellular and Molecular Life Sciences : CMLS.

[CR5] Benito E, Barco A (2010). CREB’s control of intrinsic and synaptic plasticity: implications for CREB-dependent memory models. Trends Neurosci.

[CR6] Bondy S (2016). Low levels of aluminum can lead to behavioral and morphological changes associated with Alzheimer’s disease and age-related neurodegeneration. Neurotoxicology.

[CR7] Bondy SC (2010). The neurotoxicity of environmental aluminum is still an issue. Neurotoxicology.

[CR8] Buée L, Bussière T, Buée-Scherrer V, Delacourte A, Hof P (2000). Tau protein isoforms, phosphorylation and role in neurodegenerative disorders, *Brain research*. Brain Res Rev.

[CR9] Drechsel DN, Hyman AA, Cobb MH, Kirschner MW (1992). Modulation of the dynamic instability of tubulin assembly by the microtubule-associated protein tau. Mol Biol Cell.

[CR10] Drummond E, Pires G, MacMurray C, Askenazi M, Nayak S, Bourdon M, Safar J, Ueberheide B, Wisniewski T (2020). Phosphorylated tau interactome in the human Alzheimer’s disease brain. Brain : a Journal of Neurology.

[CR11] Ebert M, Sharp P (2012). Roles for microRNAs in conferring robustness to biological processes. Cell.

[CR12] Exley C, House E, Polwart A, Esiri M (2012). Brain burdens of aluminum, iron, and copper and their relationships with amyloid-β pathology in 60 human brains. Journal of Alzheimer’s Disease : JAD.

[CR13] Feng X, Wang Z, Fillmore R, Xi Y (2014). MiR-200, a new star miRNA in human cancer. Cancer Lett.

[CR14] Ferreira PC, Piai KDA, Takayanagui AMM, Segura-Muñoz SI (2008). Aluminum as a risk factor for Alzheimer’s disease. Revista Latino Americana De Enfermagem.

[CR15] Flavell SW, Greenberg ME (2008). Signaling mechanisms linking neuronal activity to gene expression and plasticity of the nervous system. Annu Rev Neurosci.

[CR16] Frank D, Greenberg M (1994). CREB: a mediator of long-term memory from mollusks to mammals. Cell.

[CR17] Gao J, Wang W, Mao Y, Gräff J, Guan J, Pan L, Mak G, Kim D, Su S, Tsai L (2010). A novel pathway regulates memory and plasticity via SIRT1 and miR-134. Nature.

[CR18] Gong B, Vitolo OV, Trinchese F, Liu S, Shelanski M, Arancio O (2004). Persistent improvement in synaptic and cognitive functions in an Alzheimer mouse model after rolipram treatment. J Clin Investig.

[CR19] Gonzalez GA, Montminy MR (1989). Cyclic AMP stimulates somatostatin gene transcription by phosphorylation of CREB at serine 133. Cell.

[CR20] Hampel H, Blennow K, Shaw LM, Hoessler YC, Zetterberg H, Trojanowski JQ (2010). Total and phosphorylated tau protein as biological markers of Alzheimer’s disease. Exp Gerontol.

[CR21] Hou L, Dong W, Baccarelli A (2011). Environmental chemicals and microRNAs. Mutat Res.

[CR22] Idda ML, Munk R, Abdelmohsen K, Gorospe M (2018) Noncoding RNAs in Alzheimer’s disease. Wiley Interdiscip Rev RNA e1463 10.1002/wrna.1463 PMC5847280 29327503

[CR23] Izaurralde E (2015) Breakers and blockers—miRNAs at work. Science 349:380–382 10.1126/science.1260969 26206919

[CR24] Jensen PH, Hager H, Nielsen MS, Højrup P, Gliemann J, Jakes R (1999) α-Synuclein binds to tau and stimulates the protein kinase A-catalyzed tau phosphorylation of serine residues 262 and 356. J Biol Chem 10.1074/jbc.274.36.25481 10464279

[CR25] Jin N, Qian W, Yin X, Zhang L, Iqbal K, Grundke-Iqbal I, Gong C, Liu F (2013). CREB regulates the expression of neuronal glucose transporter 3: a possible mechanism related to impaired brain glucose uptake in Alzheimer’s disease. Nucleic Acids Res.

[CR26] Kandel E (2012). The molecular biology of memory: cAMP, PKA, CRE, CREB-1, CREB-2, and CPEB. Mol Brain.

[CR27] Kandimalla R, Vallamkondu J, Corgiat E, Gill K (2016). Understanding aspects of aluminum exposure in Alzheimer’s disease development. Brain Pathology (zurich, Switzerland).

[CR28] Kandimalla R, Vallamkondu J, Corgiat EB, Gill KD (2016). Understanding aspects of aluminum exposure in Alzheimer’s disease development. Brain Pathol.

[CR29] Kang H (2017) Role of microRNAs in TGF-β signaling pathway-mediated pulmonary fibrosis. Int J Mol Sci 18 10.3390/ijms18122527 PMC5751130 29186838

[CR30] Kinose Y, Sawada K, Nakamura K, Kimura T (2014). The role of microRNAs in ovarian cancer. Biomed Res Int.

[CR31] Klein G (2005). Aluminum: new recognition of an old problem. Curr Opin Pharmacol.

[CR32] Kreppel L, Hart G (1999). Regulation of a cytosolic and nuclear O-GlcNAc transferase. Role of the Tetratricopeptide Repeats, the Journal of Biological Chemistry.

[CR33] Krewski D, Yokel R, Nieboer E, Borchelt D, Cohen J, Harry J, Kacew S, Lindsay J, Mahfouz A, Rondeau V (2007) Human health risk assessment for aluminium, aluminium oxide, and aluminium hydroxide. J Toxicol Environ Health B Crit Rev 1–269 10.1080/10937400701597766 PMC2782734 18085482

[CR34] Lau P, Bossers K, Janky R, Salta E, Frigerio C, Barbash S, Rothman R, Sierksma A, Thathiah A, Greenberg D, Papadopoulou A, Achsel T, Ayoubi T, Soreq H, Verhaagen J, Swaab D, Aerts S, De Strooper B (2013). Alteration of the microRNA network during the progression of Alzheimer’s disease. EMBO Mol Med.

[CR35] Leyns C, Holtzman D (2017). Glial contributions to neurodegeneration in tauopathies. Mol Neurodegener.

[CR36] Liang Z, Liu F, Grundke-Iqbal I, Iqbal K, Gong C (2007). Down-regulation of cAMP-dependent protein kinase by over-activated calpain in Alzheimer disease brain. J Neurochem.

[CR37] Li B, Chen Y, Pang F (2020). MicroRNA-30a targets ATG5 and attenuates airway fibrosis in asthma by suppressing autophagy. Inflammation.

[CR38] Li H, Liu Q, Xue X, Lu X, Song J, He C, Hao Y, Nie J, Zhang Q, Zhao Y, Pan B, Wang L, Niu Q (2021). miR-29a/b1 regulates BACE1 in aluminum-induced Aβ deposition in vitro. ACS Chem Neurosci.

[CR39] Li J, Li R, Wu X, Hoo R, Lee S, Cheung T, Ho B, Leung G (2021). Amauroderma rugosum protects PC12 cells against 6-OHDA-induced neurotoxicity through antioxidant and antiapoptotic effects. Oxid Med Cell Longev.

[CR40] Litersky J, Johnson G, Jakes R, Goedert M, Lee M, Seubert P (1996) Tau protein is phosphorylated by cyclic AMP-dependent protein kinase and calcium/calmodulin-dependent protein kinase II within its microtubule-binding domains at Ser-262 and Ser-356. Biochem J 655–660 10.1042/bj3160655 PMC1217397 8687413

[CR41] Liu C, Wang J, Li L, Xue L, Zhang Y, Wang P (2014). MicroRNA-135a and -200b, potential biomarkers for Alzheimer’s disease, regulate β secretase and amyloid precursor protein. Brain Res.

[CR42] Lonze BE, Ginty DD (2002). Function and regulation of CREB family transcription factors in the nervous system. Neuron.

[CR43] Mandelkow EM, Mandelkow E (2012). Biochemistry and cell biology of tau protein in neurofibrillary degeneration. Cold Spring Harb Perspect Med.

[CR44] Mantamadiotis T, Lemberger T, Bleckmann SC, Kern H, Kretz O (2002) Disruption of CREB function in brain leads to neurodegeneration. Nat Genet 10.1038/ng882 11967539

[CR45] Matsuzaki K, Yamakuni T, Hashimoto M, Haque A, Shido O, Mimaki Y, Sashida Y, Ohizumi Y (2006). Nobiletin restoring beta-amyloid-impaired CREB phosphorylation rescues memory deterioration in Alzheimer’s disease model rats. Neurosci Lett.

[CR46] Peethumnongsin E, Yang L, Kallhoff-Muñoz V, Hu L, Takashima A, Pautler R, Zheng H (2010). Convergence of presenilin- and tau-mediated pathways on axonal trafficking and neuronal function. The Journal of Neuroscience : the Official Journal of the Society for Neuroscience.

[CR47] Puzzo D, Vitolo O, Trinchese F, Jacob J, Palmeri A, Arancio O (2005). Amyloid-beta peptide inhibits activation of the nitric oxide/cGMP/cAMP-responsive element-binding protein pathway during hippocampal synaptic plasticity. The Journal of Neuroscience : the Official Journal of the Society for Neuroscience.

[CR48] Rajasekaran S, Rajaguru P, Sudhakar Gandhi P (2015). MicroRNAs as potential targets for progressive pulmonary fibrosis. Front Pharmacol.

[CR49] Ratnadiwakara M, Mohenska M, Änkö M (2018). Splicing factors as regulators of miRNA biogenesis - links to human disease. Semin Cell Dev Biol.

[CR50] Sakamoto K, Karelina K, Obrietan K (2011). CREB: a multifaceted regulator of neuronal plasticity and protection. J Neurochem.

[CR51] Schmid R, Graff R, Schaller M, Chen S, Schachner M, Hemperly J, Maness P (1999). NCAM stimulates the Ras-MAPK pathway and CREB phosphorylation in neuronal cells. J Neurobiol.

[CR52] Stahl T, Falk S, Rohrbeck A, Georgii S, Herzog C, Wiegand A, Hotz S, Boschek B, Zorn H, Brunn H (2017). Migration of aluminum from food contact materials to food-a health risk for consumers? Part I of III: exposure to aluminum, release of aluminum, tolerable weekly intake (TWI), toxicological effects of aluminum, study design, and methods. Environ Sci Eur.

[CR53] Takousis P, Sadlon A, Schulz J, Wohlers I, Dobricic V, Middleton L, Lill C, Perneczky R, Bertram L (2019). Differential expression of microRNAs in Alzheimer’s disease brain, blood, and cerebrospinal fluid. Alzheimer’s & Dementia : the Journal of the Alzheimer’s Association.

[CR54] Taskén K, Skålhegg BS, Taskén KA, Solberg R, Knutsen HK, Levy FO, Sandberg M, Orstavik S, Larsen T, Johansen AK (1997). Structure, function, and regulation of human cAMP-dependent protein kinases. Adv Second Messenger Phosphoprotein Res.

[CR55] Taylor SS, Ilouz R, Zhang P, Kornev AP (2012). Assembly of allosteric macromolecular switches: lessons from PKA. Nat Rev Mol Cell Biol.

[CR56] Torreilles F, Touchon J (2002). Pathogenic theories and intrathecal analysis of the sporadic form of Alzheimer’s disease. Prog Neurobiol.

[CR57] Vasudevaraju P, Govindaraju M, Palanisamy A, Sambamurti K, Rao K (2008). Molecular toxicity of aluminium in relation to neurodegeneration. Indian J Med Res.

[CR58] Wang J, Grundke-Iqbal I, Iqbal K (2007). Kinases and phosphatases and tau sites involved in Alzheimer neurofibrillary degeneration. Eur J Neurosci.

[CR59] Wang Y, Li H, Zhang J, Han Y, Niu Q (2021). Effect of aluminum combined with ApoEε4 on tau phosphorylation and Aβ deposition. J Trace Elem Med Biol.

[CR60] Walton J (2006). Aluminum in hippocampal neurons from humans with Alzheimer’s disease. Neurotoxicology.

[CR61] Walton J (2007). An aluminum-based rat model for Alzheimer’s disease exhibits oxidative damage, inhibition of PP2A activity, hyperphosphorylated tau, and granulovacuolar degeneration. J Inorg Biochem.

[CR62] Walton J (2010). Evidence for participation of aluminum in neurofibrillary tangle formation and growth in Alzheimer’s disease. Journal of Alzheimer’s Disease : JAD.

[CR63] Weingarten M, Lockwood AH, Kirschner H (1975). A protein factor essential for microtubule assembly. Proc Natl Acad Sci USA.

[CR64] Wilcock GK, Esiri MM (1982). Plaques tangles and dementia. A quantitative study. J Neurol Sci 56: 343–356. J Neurol Sci.

[CR65] Wu Q, Lu R, Li J, Rong L (2017). MiR-200a and miR-200b target PTEN to regulate the endometrial cancer cell growth in vitro. Asian Pac J Trop Med.

[CR66] Xie S, Jin N, Gu J, Shi J, Sun J, Chu D, Zhang L, Dai C, Gu J, Gong C, Iqbal K, Liu F (2016). O-GlcNAcylation of protein kinase A catalytic subunits enhances its activity: a mechanism linked to learning and memory deficits in Alzheimer’s disease. Aging Cell.

[CR67] Yamamoto-Sasaki M, Ozawa H, Saito T, Rösler M, Riederer P (1999). Impaired phosphorylation of cyclic AMP response element binding protein in the hippocampus of dementia of the Alzheimer type. Brain Res.

[CR68] Yasui M, Kihira T, Ota K (1992). Calcium, magnesium and aluminum concentrations in Parkinson’s disease. Neurotoxicology.

[CR69] Zhang H, Zhang SB, Zhang QQ, Liu M, He XY, Zou Z, Sun HJ, You ZD, Shi XY (2013). Rescue of cAMP response element-binding protein signaling reversed spatial memory retention impairments induced by subanesthetic dose of propofol. CNS Neurosci Ther.

[CR70] Zhang Q, Liu W, Lu G (2017). miR-200a-3p promotes b-Amyloid-induced neuronal apoptosis through down-regulation of SIRT1 in Alzheimer’s disease. J Biosci.

